# In transition: current health challenges and priorities in Sudan

**DOI:** 10.1136/bmjgh-2019-001723

**Published:** 2019-08-21

**Authors:** Esmita Charani, Aubrey J Cunnington, AlaEldin H A Yousif, Mohammed Seed Ahmed, Ammar E M Ahmed, Souad Babiker, Shahinaz Badri, Wouter Buytaert, Michael A Crawford, Mustafa I Elbashir, Kamal Elhag, Kamal E Elsiddig, Nadey Hakim, Mark R Johnson, Alexander D Miras, Mohamed O Swar, Michael R Templeton, Simon David Taylor-Robinson

**Affiliations:** 1 Department of Medicine, Faculty of Medicine, Imperial College London, London, UK; 2 Department of Medicine, Imperial College London, London, UK; 3 Department of Medicine, University of Khartoum, Khartoum, Sudan; 4 Department of Medicine, Ahfad University for Women, Omdurman, Sudan; 5 Department of Pathology, School of Medicine, Ahfad University for Women, Omdurman, Sudan; 6 Department of Medicine, St. Mary's Hospital Campus, Imperial College London, London, UK

**Keywords:** nutrition, maternal health, malaria, diabetes, cancer, hygiene, surgery

## Abstract

A recent symposium and workshop in Khartoum, the capital of the Republic of Sudan, brought together broad expertise from three universities to address the current burden of communicable and non-communicable diseases facing the Sudanese healthcare system. These meetings identified common challenges that impact the burden of diseases in the country, most notably gaps in data and infrastructure which are essential to inform and deliver effective interventions. Non-communicable diseases, including obesity, type 2 diabetes, renal disease and cancer are increasing dramatically, contributing to multimorbidity. At the same time, progress against communicable diseases has been slow, and the burden of chronic and endemic infections remains considerable, with parasitic diseases (such as malaria, leishmaniasis and schistosomiasis) causing substantial morbidity and mortality. Antimicrobial resistance has become a major threat throughout the healthcare system, with an emerging impact on maternal, neonatal and paediatric populations. Meanwhile, malnutrition, micronutrient deficiency and poor perinatal outcomes remain common and contribute to a lifelong burden of disease. These challenges echo the United Nations (UN) sustainable development goals and concentrating on them in a unified strategy will be necessary to address the national burden of disease. At a time when the country is going through societal and political transition, we draw focus on the country and the need for resolution of its healthcare needs.

**Summary box**

- The Republic of Sudan is undergoing dramatic political and societal changes which have potential to both improve and harm the health of the population.
- Poverty-associated endemic infectious diseases, nutritional deficiencies and poor perinatal outcomes cause a huge burden of ill health.
- An increasing burden of chronic non-communicable disease, and communicable diseases pose major challenges for to the healthcare system.
- Antimicrobial resistance is widespread and threatens all aspects of the healthcare system.
- Improvements in population health require improvements in multi-sector infrastructure and better data to prioritise the use of resources.

## Introduction

**Figure 1:**
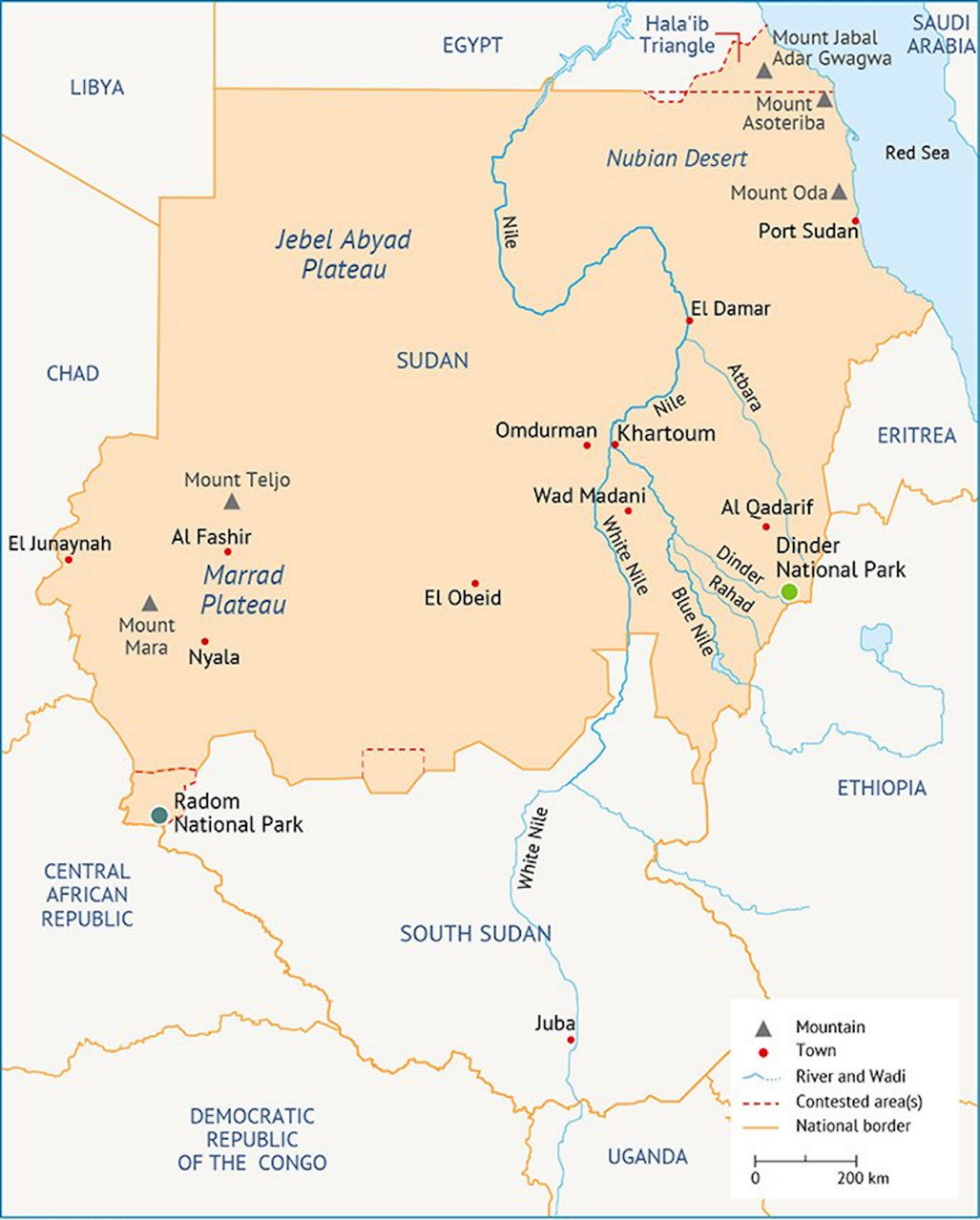
Map of Sudan, reproduced from https://fanack.com/sudan/geography/, site accessed on 8 July 2019

Sudan has long held a fascination to the outsider, right from the Biblical days of the Kingdoms of Kush and Nubia, through the Ottoman Empire to the Victorian era, where there was joint rule as a Condominium by the United Kingdom and Egypt, until independence in 1956. At independence, the country was considered the breadbasket of Africa and irrigation schemes made the country rich in cotton and other agricultural products. However, decades of mismanagement, the harsh climate and political difficulties have meant that this is now all but vestigial. Notably, a long-standing war of separation between the South and the North led to the splitting of Africa’s largest country, when the Republic of South Sudan was born after a UN-approved referendum in 2011^1^ (figure 1). The remainder of the country has had further troubles with intermittent famine in both the East and West, long-running conflicts in Darfur (the provinces that make up the western border with Chad) and similar difficulties along the Eritrean border on the Red Sea Coast. These conflicts have added a considerable and constant stream of refugees and displaced people seeking safety in distant parts of the country with the capital Khartoum swelling from a town of barely 150 000 people at independence to a metropolis of 5 million people today.^2^ At a time when political change has been affected by popular pressure, and when the politics in Sudan is undergoing transition, focus on Sudan and its medical issues is particularly pertinent.

Though steady progress has been made, communicable diseases are still estimated to cause 30% of deaths in Sudan (Institute for Health Metrics and Evaluation data, see table 1), and many challenges remain. As with other countries of the Sahel, schistosomiasis, leishmaniasis and malaria are common, the former having first been noticed in Biblical days when Nubia was considered the ‘land of menstruating men’, owing to haematuria from *Schistosoma haematobium* infection.^3^ More recently, alarming rates of antimicrobial resistance (AMR) have been noted, which threaten to sustain or even increase the communicable disease burden. Gaps in infrastructure, such as lack of appropriate access to laboratories, limited resources, including inadequate workforce, and the fragility of the healthcare system, contribute to poor implementation of programmes which, in turn, undermines efforts to tackle diseases. Access to safe water is central to communicable disease control and despite encouraging trends in the right direction nationally,^4^ inadequate water, sanitation and hygiene still contribute to a high burden of communicable diseases, including diarrhoeal deaths.^5^ Adequate access to clean water and sanitation are also essential for limiting the spread of pathogens, reducing the need for antibiotics and preventing the emergence and spread of AMR.^6^ As with almost all societies around the world, Sudanese people are becoming more obese with type 2 diabetes and its complications becoming an increasing problem, and non-communicable diseases are accounting for an ever-greater burden on the health system (table 1).

**Table 1:** Trends in the estimated burden of selected diseases in Sudan from 2007 to 2017 (data from https://vizhub.healthdata.org/gbd-compare/)

Selected causes	Estimated deaths (change from 2007)	Estimated deaths/100, 000 (change from 2007)	Estimated disability-adjusted life year (DALYs) (change from 2007)	Estimated DALYs/100 000 (change from 2007)
Neonatal	25 200 (−24%)	63 (−40%)	2 300 000 (−22%)	5800 (−38%)
Maternal	2400 (−22%)	6 (−40%)	140 000 (−22%)	344 (−38%)
Lower respiratory tract infections	9400 (−34%)	23 (−48%)	620 000 (−41%)	1500 (−54%)
Diarrhoea	8200 (−46%)	20 (−57%)	780 000 (−42%)	1900 (−55%)
Malaria	2600 (−13%)	6 (−32%)	180 000 (−16%)	450 (−33%)
Ischaemic heart disease	41 700 (+11%)	104 (−12%)	960 000 (+12%)	2400 (−12%)
Stroke	18 200 (+0.8%)	45 (−20%)	480 000 (−1%)	1200 (−21%)
Chronic obstructive pulmonary disease	3400 (+12%)	8 (−15%)	150 000 (+20%)	360 (−5%)
Diabetes	3200 (+20%)	8 (−5%)	200 000 (+30%)	500 (+2.4%)
Chronic kidney disease	3200 (+8%)	8 (−12%)	140 000 (+3%)	340 (−20%)

In November 2018, the first scientific symposium was held between University of Khartoum, Ahfad University for Women and Imperial College London. The goal of the 3-day symposium was to address the burden of disease in Sudan by enhancing academic and scientific collaboration. Stakeholder discussions identified three broad themes for the symposium, focusing on areas in which the universities have particular expertise: communicable diseases, non-communicable diseases, women’s and children’s health and nutrition. This first exploratory symposium brought together academics, clinicians and young researchers from the three institutions. Through a series of keynote presentations, interactive focus groups and workshops, knowledge and ideas were exchanged, complementary research priorities were defined and strategies for future partnerships discussed, all with the goal of tackling key challenges facing the Sudanese healthcare system. This article synthesises the content of these discussions with other published evidence in order to inform and highlight areas in which collaborative efforts may bring particular benefit.

## Communicable diseases

### Water and sanitation

Despite encouraging trends in the right direction when it comes to water and sanitation coverage nationally in Sudan, diarrhoeal deaths attributable to lack of adequate water, sanitation and hygiene remain a considerable challenge today.^4^ In 2015, 26% of people nationally in Sudan were reported to practice open defecation and a further 30% relied on unimproved forms of sanitation, while access to basic and safely managed drinking water was highly variable, ranging from 30% in North Darfur to 95% in Khartoum.^4^ In many parts of the country, inhabitants rely on finding water outside their home, due to the lack of piped water distribution networks. The sources of water are often of dubious chemical and microbial quality. Because of the scarcity of groundwater resources,^7^ most people rely on surface water sources or shallow groundwater wells with a high risk of contamination. Water can also become contaminated because of insecure water transportation and storage practices on the way to and within the home.^8^ Outside the home, water and sanitation in schools and health facilities are also widely inadequate, especially in rural remote areas.^9^ Added to the diarrhoeal burden is the high prevalence of neglected tropical diseases in certain parts of Sudan, such as schistosomiasis in East and South Darfur States,^10^ the prevention of which also relies on improvements in household and community water supply and sanitation conditions to complement mass drug administration efforts.^11 12^

The precarious water and sanitation conditions are exacerbated further by the country’s exposure to extreme climate events.^13^ Around 4.6 million people in Sudan are food insecure, but these numbers rise steeply during drought years.^14^ Drought is also thought to trigger political conflicts and social instability, which leads to a vicious circle of degrading living conditions and poverty.^15^ Future water management initiatives designed to address food and energy insecurity in Sudan, such as new irrigation schemes or dams, should be mindful of the lessons of past projects that were linked to the spread of certain diseases, such as the experience of the Gezira Scheme and schistosomiasis in Sudan several decades ago.^16^ Finally, flooding occurs regularly during the rainy seasons, in particular along the borders of the Nile.^16^ This is associated with flood-induced public health risks and disease outbreaks such as hepatitis.^17^

Addressing these challenges will require increased data collection and ongoing monitoring of the disease burdens and water and sanitation coverage levels, especially in rural regions, and ultimately increased investment in improved water and sanitation facilities and infrastructure. These will require internal political commitment and continued support from external sources such as UNICEF.^4^ An additional significant part of the problem in Sudan is that there is a shortage of qualified personnel to operate and maintain the necessary water and sanitation infrastructure, and skilled graduates are lost to other sectors, or they are unwilling to live and work in the remote areas where the problems are most serious.^4^ Future efforts aimed towards minimising the health outcomes associated with reliance on poor quality water and poor sanitation conditions must therefore combine technical solutions with sustainable human resource strategies.

### Malaria

*Plasmodium falciparum* malaria is endemic, with an estimated 41 million people at risk of malaria and 1.5 million new cases diagnosed in 2017, representing 35% of the malaria burden in the whole Eastern Mediterranean region.^18^ The intensity of transmission ranges from extremely low in Khartoum and the north, to very high in the southwest of the country,^18^ with seasonal and unstable patterns dependent on rainfall. In urban settings and irrigated areas transmission can also occur year-round. Naturally acquired immunity is low in much of the population, meaning that all age groups are at risk of symptomatic infection.^19^ Consequently, there is a risk for malaria epidemics following heavy rains or floods and rapid increases in mosquito populations. In common with many other countries, national malaria surveys in Sudan showed a decline in national parasite prevalence from 3.7% in 2000 to 1.8% in 2009. However, subsequent increases to 3.3% in 2012 and 5.4% in 2016 were concerning.^20^ ^21^

Reductions in funding for malaria control, suboptimal coverage of long-lasting insecticide-treated nets and lower rates of malaria testing and reporting than most other African countries all create challenges for Sudan to meet its goal of 20%–40% reduction in malaria by 2020.^18^ A recent change in first-line treatment for malaria, from artemether sulphadoxine-pyrimethamine (which had >10% treatment failure rates) to artemether-lumefantrine and dihydroartemisinin-piperaquine may go some way to restoring progress.^18^

*Plasmodium vivax* is an emerging pathogen in Sudan, estimated by WHO to account for around 10% of malaria cases nationally,^18^ although recent reports indicate up to 40% of hospital cases in the White Nile region^22^ and a third of paediatric severe malaria cases in Khartoum^23^ are due to *P. vivax*. The prevalence of *Plasmodium ovale* and *Plasmodium malariae* are unknown. Treatment of *P. vivax* requires additional radical cure of liver hypnozoites, otherwise relapses can ensue.^24^ Unfortunately, the main drug for this purpose, primaquine, can cause severe haemolysis in individuals with glucose-6-phosphate dehydrogenase (G6PD) deficiency. The prevalence of this genetic trait is poorly characterised in Sudan^25^ and so an increase in *P. vivax* cases without infrastructure for routine assays of G6PD function creates dilemmas for treatment. Furthermore, primaquine has an additional attractive property for use in Sudan—it kills *P. falciparum* gametocytes—and mass drug administration of primaquine (with artemisinin combination therapy) to whole populations has been proposed as an intervention to reduce malaria transmission.^26^ This approach might be particularly effective in regions of Sudan with short transmission seasons linked to seasonal rainfall. Therefore, characterising the prevalence of G6PD deficiency throughout Sudan seems to be an important priority for malaria control.

### Schistosomiasis

While malaria may cause the greatest acute health burden, schistosomiasis is believed to be the most prevalent parasitic infection. Indeed, one of the first ever treatment trials for this disease was conducted in Khartoum.^27^ Both *Schistosoma haematobium* and *S*chistosoma* mansoni* are present, and being water-transmitted infections, their epidemiology is strongly associated with proximity and exposure to natural freshwater sources.^10 28^ National control efforts, through mass drug administration, have previously been restricted by a paucity of epidemiological data to support their targeting.^29^ A recent nationwide survey of over 100 000 school children has now provided compelling evidence to support introduction of mass drug administration programmes and identified modifiable risk factors. The overall prevalence of *S. haematobium* infection was 5.2%, and *S. mansoni* 0.06%, but ranged much higher in East Darfur state where *S. haematobium* prevalence reached over 25%.^10^ Previous reports have identified even higher focal prevalence; for example in some schools of the White Nile region, 46.5% of sampled children were infected (45% *S*. *haematobium*, 5.9% *S*. *mansoni* and 4.4% mixed infection).^28^ Frequent contact with bodies of water was a major risk factor, whereas using latrines at home and school was protective.^30^

### Leishmaniasis

Cutaneous leishmaniasis is widespread with 93% of the population considered at risk, but visceral disease is more geographically restricted to southern, eastern and central regions, with about 25% of the population considered at risk.^30 31^ Despite the large number of individuals at risk, the incidence is relatively low, and the WHO estimates there are approximately 3000 cases per year of visceral leishmaniasis and 3500 of cutaneous leishmaniasis.^31^ There have historically been outbreaks of cases associated with population movements from endemic parts of the country into previously non-endemic regions, although densities of both vector sandflies and reservoir animals (Nile rats) are additional determinants of incidence.^31^ Cutaneous leishmaniasis in Sudan is caused by *Leishmania major*, whereas visceral disease is caused *by Leishmania donovani*, with recent serological studies indicating that there may be different strains of *L. donovani* in distinct geographical regions.^30^ Although leishmaniasis is a notifiable disease in Sudan, the burden of visceral disease may be underestimated, because definitive diagnosis is relatively difficult. Gold-standard diagnosis by detection of the parasite in tissue specimens is not possible in most rural health facilities. Rapid serological tests provide an alternative, but the rK39 antigen-based test has proven less sensitive in Sudan than in the Indian subcontinent^32^ and the test has a relatively short shelf-life which can make it uneconomical to keep supplies outside of major centres.^33^ Ongoing work has been seeking to address the need for cheap, sensitive, specific and sustainable rapid diagnostics for Sudanese visceral leishmaniasis, which will help to better define disease burden, identify cases for prompt treatment and potentially allow confirmation of post-treatment cure.^32–34^

### Antimicrobial resistance

Prevention strategies are key to the control of the emergence and spread of AMR. These include vaccination, access to water and sanitation (discussed earlier) and effective infection prevention and control procedures. Inadequacies across the healthcare system hamper prevention efforts. Furthermore, there is limited published literature on AMR surveillance or antibiotic use from Sudan.^35^ Recent reports document worryingly high rates of multi-drug resistance in some hospital settings.^35 36^ Up to 92% of urinary *Escherichia coli* clinical isolates have been reported to be multi-drug resistant.^37^ In a study in Khartoum investigating the knowledge and perception of hospital doctors on the importance and causes of spread of AMR, while inappropriate antibiotic use was widely accepted as one of the causes of AMR, inadequate handwashing was only recognised as a factor for its spread by one third of the 350 physicians surveyed.^38^ There is widespread antibiotic prescribing by healthcare professionals in hospitals, with up to 65% of patients receiving antibiotics, including 45% of patients with malaria, inappropriately prescribed antibiotic.^36^ Inappropriate surgical prophylaxis and postoperative use are major sources of antibiotic consumption.^39 40^ One study, investigating antibiotic prophylaxis in a teaching hospital in Khartoum found 97% of patients undergoing surgery had extended duration of surgical prophylaxis.^39^

Infections in maternal and neonatal and paediatric surgical pathways are an additional concern. Female genital mutilation is widely practiced, and this practice leads to severe complications during delivery, often requiring deinfibulation, carried out by non-medical healthcare professionals.^41^ As a result, the maternal sepsis rate is among the highest in this part of the world, presenting as one of the key causes of morbidity and mortality after childbirth.^42 43^ Acute malnutrition is another major risk factor for sepsis, primarily in infants and children, and Sudan has one of the highest rates of malnutrition in the Middle East and North Africa. Malnutrition in children under the age of 5 years is reported to be 16% with very high rates of infection reported in this population.^44^

Governmental support and commitment for initiatives to target AMR is are necessary for implementation and sustainability. In July 2017, Sudan became one of the first countries in Africa to draft a National Action Plan (NAP) for AMR.^45^ Drafted in collaboration between WHO and the Sudanese Ministries of Agriculture and Forestry, Health, Animal Resources and Fisheries, this NAP showed a commitment from the Government to the one health approach. It is imperative to make the most of this initiative and commitment and develop sustainable pathways to solve the burgeoning challenge of AMR. Through existing networks, collaborations and shared experiences there are many opportunities for bi-directional learning from existing successful programmes in other low- and middle- income countries (LMICs). The key issues to address in relation to AMR are to^1^: ensure the one health approach to implementing the NAP is carried out to address the human, animal and environmental (eg, water and sanitation) drivers for AMR^2^; develop systems for surveillance and research to optimise antibiotic use across the healthcare economy^3^; raise awareness and understanding of AMR^4^; understand the contextual factors that can be addressed to ensure the sustainable implementation of the NAP^5^; through a more broad perspective understand the cultural behaviours, including FGM, that contribute to poor infection-related outcomes for vulnerable maternal and paediatric populations.

## Non-communicable diseases

### Diabetes and obesity

Despite the division from South Sudan in 2011, Sudan remains one of the largest countries in Africa with a population just over 40 million.^46^ The prevalence of diabetes in the northern part of the country is estimated to be around 20% of the population.^47^ The increase in incidence of diabetes can be attributed to features of urbanisation such as an increase in the prevalence of obesity, a lack of physical activity through reduced manual labour and an increase in consumption of refined carbohydrates and high sugar intake.^48–50^ In one study evaluating attendance to clinic for type 2 diabetes, 85% of patients were found to have poor control and a high prevalence of complications was observed.^51^ In this population hypertension was found in 40%, myocardial infarction in 6%, peripheral neuropathy in 68%, retinopathy in 73% and diabetic foot in 13%.^51^ Hypercholesterolaemia and hypertriglyceridaemia were noted in 60% and 33%, respectively, while low levels of high-density lipoprotein cholesterol were found in 53%. In a separate study, over one-third of patients with diabetic retinopathy were found to have nephropathy.^50^ Thus, a high prevalence of diabetes-associated renal disease is likely in Sudan, and it is particularly concerning because the country has very few resources to deal an impending epidemic of end-stage renal disease. The emerging data showing widespread AMR have particularly grim implications for those who will need frequent hospital treatment for complications of diabetes, obesity and renal failure.

### Cancer

Like other major non-communicable diseases, the prevalence and burden of cancer in Sudan appear to be increasing.^52^ The most common cancers in females are breast cancer, followed by uterine cervical cancer and ovarian cancer.^52 53^ In males, the most common malignancies were prostate cancer, leukaemia, lymphoma and colorectal cancer.^52^ In common with many other African countries, the high burden of some infections, which promote tumour development, such as human papilloma virus, Epstein Barr virus and schistosomiasis, contributes to the cancer burden.^52^ Despite the expanding population of more than 40 million, there are only two major cancer centres in the country: Khartoum Oncology Specialized Center in the capital, and the National Cancer Institute in Gezira State, central Sudan. These limited resources have led to the development of other smaller facilities addressing the increasing numbers of breast cancer in Sudan, such as the Khartoum Breast Care Center, a centre of excellence, but without a radiation therapy unit. Though a central cancer registry was first founded in the 1960s and was reinstated in 2009^53^ the lack of a comprehensive national surveillance system for cancer has held back better understanding of the epidemiology and outcomes of cancer in the country. Furthermore, relatively little is known about the underlying biology of some of the most common cancers in Sudan, although a few studies have hinted that there may be unique features in this population.^52 54^ To promote better understanding of the biological basis of cancers in Sudan, and ultimately improve their treatment, there is a need for a national biobank as a central repository for tissues and other samples from cancer patients.

## Maternal and child health

While many outsiders equate Sudan with traditional practices of female circumcision and accompanying high maternal and neonatal mortality rates, childhood malnutrition remains a significant issue not only in displaced people from war and conflict but also in many rural communities, where there is limited access to medical care. Rates of adverse perinatal outcome and associated disability are high in Sudan, as in many countries in sub-Saharan Africa.^55 56^ Maternal mortality is often associated with infection, haemorrhage, pre-eclampsia or obstructed labour, while preterm birth accounts for the majority of infant deaths.^56^ Despite this, variations in perinatal epidemiology are poorly described in the country. For example, although preterm birth is stated to be the most important cause of infant mortality, most data are estimates, based on birth weight which may be confounded by fetal growth restriction.^57^

A 2013 publication described Sudan as having ‘made insufficient progress to achieve Millenium Development Goal 4 and having levels of child and infant mortality that are among the highest in the region and the world’.^58^ The current infant mortality rate is 60 per 1000 live births and the under-5-year mortality rate is 82 deaths per 1000 live births.^58 59^ The neonatal mortality rate is also high, ranging from 34 to 47 per 1000 births^59^ with a maternal mortality rate of 311 deaths/100 000 live births.^58^ The preterm birth rate in Sudan is estimated to be 13.3%<37 weeks of gestation (everywomeneverychild.org). Accurately defining perinatal outcomes and relating them both to events in the pregnancy and to the eventual infant outcomes is a critical step in the development of interventions to improve perinatal and population health.

Maternal health and nutrition are also important for foetal neurological development, and the high prevalence of iodine deficiency in Sudan^60^ is likely to have a significant impact on neurodevelopmental outcomes. Populations living inland, distant from the sea, are at particularly high risk, while there is little or no iodine deficiency to be seen in the fishing villages of the same regions.^61^ Data from Sudan describing deficits of docosahexaenoic acid (DHA) (an omega-3 fatty acid that is a primary structural component of the human brain, cerebral cortex, skin and retina) in maternal and cord blood and breast milk in a population at high risk due to iodine deficiency prompt the thought that the coexistence of iodine and DHA deficiency may be extremely common in inland areas of the country.^62^ Both DHA and iodine coexist at their richest in the marine food web^63^ and deficiencies of either can impair foetal and infant brain development.^64^ While the situation has improved in some affected regions, iodine deficiency remains widespread and attention to the DHA requirements may also be required for optimal developmental outcomes.

## Conclusion

This paper provides a summary of the proceedings of a 3-day scientific symposium and does not represent a systematic review of the existing research in the themes included. However, the symposium brought together extensive academic and clinical expertise and provided a unique perspective on the current challenges and gaps in the Sudanese healthcare system.

The burden of disease in Sudan is affected by poverty and is complicated by geography, politics, armed conflict and not infrequently, mismanagement. At a time when the political landscape is in transition, the burden of communicable disease in the country remains high, and non-communicable diseases look set to increase. The problems, however, are not all intractable. The symposium and workshops held in Khartoum in November 2018 highlighted strategies to improve the situation with relatively limited resources. Many of these have the common theme of collecting high quality and nationally representative data to better understand the problems and prioritise the use of scarce funding, and the need for achieving governmental support to implement improvements in healthcare.

The WHO Sustainable Development Goals should be achievable despite previously poor implementation and organisation of WHO directives. The developed world needs to work with Sudan, not only at a governmental level but also through professional bodies to support the well-qualified healthcare workforce and foster capacity building for research and development through partnerships with external funders and academic institutions across the world. There are also opportunities for bi-directional learning from other LMICs. The Khartoum conference highlighted opportunities for collaborations and workforce development to engender sustained change. The current situation in Sudan and how it unfolds will be critical in determining the evolution of healthcare distribution and also engineering societal change in the future of the country. We remain hopeful that global politics does not hinder the fundamental transformations that are needed for the current and future health of the nation.

## References

[1] Cockett R Darfur and the failure and division of an African state. Sudan: Yale University Press, 2016.

[2] United Nations Sudan country profile. Available: https://www.imuna.org/resources/country-profiles/sudan [Accessed Jul 25 2019].

[3] Kloos H, David R The paleoepidemiology of schistosomiasis in ancient Egypt. Human Ecology 2002;9:14–25.

[4] WHO/UNICEF Joint Monitoring Programme Progress on drinking water, sanitation and hygiene 2017 World Health Organisation; 2017.

[5] Berger S Infectious diseases of Sudan and South Sudan. Gideon informatics, 2017

[6] Bürgmann H, Frigon D, H Gaze W, et al Water and sanitation: an essential battlefront in the war on antimicrobial resistance. FEMS Microbiol Ecol 2018;94 10.1093/femsec/fiy101 29878227

[7] Hamad OE-T, El-Battahani A Sudan and the Nile Basin. Aquat Sci 2005;67:28–41. 10.1007/s00027-004-0767-9

[8] Yassin K, Ibrahim R Quality aspects of manually transported drinking water in the Outskirts of Khartoum state. Univ Khartoum Eng J 2014;4:58–63.

[9] United Nations Global Analysis and Assessment of Sanitation and Drinking Water(GLAAS) 2015. Available: https://www.who.int/water_sanitation_health/publications/glaas_strategy.pdf?ua=1 [Accessed Jul 25 2019].

[10] Cha S, Hong S-T, Lee Y-H, et al Nationwide cross-sectional survey of schistosomiasis and soil-transmitted helminthiasis in Sudan: study protocol. BMC Public Health 2017;17:1–10. 10.1186/s12889-017-4719-4 28899362 PMC5596840

[11] Lee Y-H, Jeong HG, Kong WH, et al Reduction of urogenital schistosomiasis with an integrated control project in Sudan. PLoS Negl Trop Dis 2015;9:e3423 10.1371/journal.pntd.0003423 25569278 PMC4288734

[12] Grimes JET, Croll D, Harrison WE, et al The roles of water, sanitation and hygiene in reducing schistosomiasis: a review. Parasit Vectors 2015;8 10.1186/s13071-015-0766-9 PMC4377019 25884172

[13] Gizaw MS, Gan TY Impact of climate change and El Niño episodes on droughts in sub-Saharan Africa. Clim Dyn 2017;49:665–82. 10.1007/s00382-016-3366-2

[14] Food and Agricultiure Organization of the United Nations Early warning early action report on food security and agriculture. Rome; 2018.

[15] Selby J, Hoffmann C Beyond scarcity: rethinking water, climate change and conflict in the Sudans. Global Environmental Change 2014;29:360–70. 10.1016/j.gloenvcha.2014.01.008

[16] Fenwick A Waterborne infectious diseases--could they be consigned to history? Science 2006;313:1077–81. 10.1126/science.1127184 16931751

[17] Mudawi HM Epidemiology of viral hepatitis in Sudan. Clin Exp Gastroenterol 2008;1:9–13. 10.2147/CEG.S3887 21677820 PMC3108625

[18] World Health Organisation Who world malaria report 2018. Geneva World Health Organisation; 2018.

[19] Theander TG Unstable malaria in Sudan: the influence of the dry season. malaria in areas of unstable and seasonal transmission. lessons from Daraweesh. Trans R Soc Trop Med Hyg 1998;92:589–92. 10.1016/s0035-9203(98)90775-1 10326097

[20] Sudan federal Ministry of health. annual health statistics reports (2014). Malaria indicator survey 2012-2013 in the Republic of Sudan. Available: http://ghdx.healthdata.org/record/sudan-national-malaria-indicator-survey-2012-2013 [Accessed Jul 25 2019].

[21] Suliman MMA, Hamad BM, Albasheer MMA, et al Molecular Evidence of High Proportion of Plasmodium vivax Malaria Infection in White Nile Area in Sudan. J Parasitol Res 2016;2016(1-2, supplement):2892371:1–4. 10.1155/2016/2892371 27980861 PMC5131248

[22] Hashim HA, Ali EMA Pattern of malaria in hospitalized children in Khartoum state. Sudan J Paediatr 2017;17:35–41. 10.24911/SJP.2017.2.4 29545663 PMC5845454

[23] Phillips MA, Burrows JN, Manyando C, et al Malaria. Nat Rev Dis Primers 2017;3 10.1038/nrdp.2017.50 28770814

[24] Howes RE, Piel FB, Patil AP, et al G6Pd deficiency prevalence and estimates of affected populations in malaria endemic countries: a geostatistical model-based MAP. PLoS Med 2012;9:e1001339 10.1371/journal.pmed.1001339 23152723 PMC3496665

[25] John CC Primaquine plus artemisinin combination therapy for reduction of malaria transmission: promise and risk. BMC Med 2016;14:4–6. 10.1186/s12916-016-0611-9 27039396 PMC4818907

[26] Adeel AA When history was made in Khartoum civil Hospital: first introduction of chemotherapy for schistosomiasis. Sudan J Paediatr 2015;15:80–99. 27493441 PMC4958668

[27] Ismail HAHA, Hong S-T, Babiker ATEB, et al Prevalence, risk factors, and clinical manifestations of schistosomiasis among school children in the white Nile River Basin, Sudan. Parasit Vectors 2014;7:1–11. 10.1186/s13071-014-0478-6 25312470 PMC4200116

[28] Cha S, Elhag MS, Lee YH, et al Epidemiological findings and policy implications from the nationwide schistosomiasis and intestinal helminthiasis survey in Sudan (March 2019). Available: https://ssrn.com/abstract=3310618 [Accessed Jul 25 2019]. 10.1186/s13071-019-3689-z PMC6728938 31488219

[29] World Health Organization Neglected tropical diseases. cutaneous leishmaniasis in Sudan. Available: http://www.emro.who.int/neglected-tropical-diseases/countries/cl-sudan.html [Accessed Jul 25 2019].

[30] Mukhtar M, Abdoun A, Ahmed AE, et al Diagnostic accuracy of rK28-based immunochromatographic rapid diagnostic tests for visceral leishmaniasis: a prospective clinical cohort study in Sudan. Trans R Soc Trop Med Hyg 2015;109:594–600. 10.1093/trstmh/trv060 26246251

[31] Mahamoud A, Osman HA, Abass EM, et al Identification of an area predominantly endemic for childhood and adolescent visceral leishmaniasis in central Sudan. Acta Trop 2018;178:142–7. 10.1016/j.actatropica.2017.11.010 29183852

[32] Osman HA, Mahamoud A, Abass EM, el Harith A, et al Local production of a liquid direct agglutination test as a sustainable measure for control of visceral leishmaniasis in Sudan. Am J Trop Med Hyg 2016;94:982–6. 10.4269/ajtmh.15-0574 26976890 PMC4856630

[33] Marlais T, Bhattacharyya T, Singh OP, et al Visceral leishmaniasis IgG1 rapid monitoring of cure vs. relapse, and potential for diagnosis of post kala-azar dermal leishmaniasis. Front Cell Infect Microbiol 2018;8:1–10. 10.3389/fcimb.2018.00427 30619774 PMC6300496

[34] Abass A, Ahmed M, Ibrahim I, et al Bacterial resistance to antibiotics: current situation in Sudan. J Adv Microbiol 2017;6:1–7. 10.9734/JAMB/2017/36715

[35] Mustafa I The patterns of medicines use in Sudan: a cross sectional study at National health insurance fund setting, 2012 Available: http://apps.who.int/medicinedocs/documents/s20953en/s20953en.pdf [Accessed Jul 25 2019].

[36] Ibrahim ME, Bilal NE, Hamid ME Increased multi-drug resistant Escherichia coli from hospitals in Khartoum state, Sudan. Afr Health Sci 2012;12:368–75. 23382754 10.4314/ahs.v12i3.19 PMC3557680

[37] Kheder S Physicians' knowledge and perception of antimicrobial resistance: a survey in Khartoum state hospital settings. Br J Pharm Res 2013;3:347–62. 10.9734/BJPR/2013/2117

[38] Elbur AI, Yousif MAER, Elsayed ASA, et al An audit of prophylactic surgical antibiotic use in a Sudanese teaching hospital. Int J Clin Pharm 2013;35:149–53. 10.1007/s11096-012-9719-y 23135836

[39] Elbur AI, M A Y, El-Sayed ASA, et al Prophylactic antibiotics and wound infection. J Clin Diagn Res 2013;7:2747–51. 10.7860/JCDR/2013/6409.3751 24551629 PMC3919408

[40] Banks E, Meirik O, Farley T, et al Female genital mutilation and obstetric outcome: who Collaborative prospective study in six African countries. Lancet 2006;367:1835–41. 10.1016/S0140-6736(06)68805-3 16753486

[41] Ahmed MI, Alsammani MA, Babiker RA Puerperal sepsis in a rural hospital in Sudan. Mater Sociomed 2013;25:19 10.5455/msm.2013.25.19-22 23678336 PMC3633386

[42] Mohammed AA, Elnour MH, Mohammed EE, et al Maternal mortality in Kassala State - Eastern Sudan: community-based study using Reproductive age mortality survey (RAMOS). BMC Pregnancy Childbirth 2011;11:102 10.1186/1471-2393-11-102 22171988 PMC3260097

[43] Mahgoub HM, Adam I Morbidity and mortality of severe malnutrition among Sudanese children in new Halfa Hospital, eastern Sudan. Trans R Soc Trop Med Hyg 2012;106:66–8. 10.1016/j.trstmh.2011.09.003 22023885

[44] Essack SY, Desta AT, Abotsi RE, et al Antimicrobial resistance in the who African region: current status and roadmap for action. J Public Health 2017;39:8–13. 10.1093/pubmed/fdw015 PMC5939661 26944074

[45] United Nations World Population Prospects, 2017 Available: https://population.un.org/wpp/[Accessed Jul 25 2019].

[46] Elmadhoun WM, Noor SK, Ibrahim AAA, et al Prevalence of diabetes mellitus and its risk factors in urban communities of North Sudan: population-based study. J Diabetes 2016;8:839–46. 10.1111/1753-0407.12364 26663723

[47] El-Sayed EF, Awadalla H, Noor SK, et al Sugar intake in Sudanese individuals was associated with some features of the metabolic syndrome: population based study. Diabetes Metab Syndr 2018;12:245–50. 10.1016/j.dsx.2017.09.001 29050917

[48] Ali YA, Almobarak AO, Awadalla H, et al Obesity among Sudanese adults with diabetes: a population-based survey. Ann Transl Med 2017;5 10.21037/atm.2017.05.11 PMC5497089 28706920

[49] Khalil S, Almobarak AO, Awadalla H, et al Low levels of physical activity in Sudanese individuals with some features of metabolic syndrome: population based study. Diabetes Metab Syndr 2017;11 Suppl 2:S551–S554. 10.1016/j.dsx.2017.04.003 28420573

[50] Awadalla H, Noor SK, Elmadhoun WM, et al Diabetes complications in Sudanese individuals with type 2 diabetes: overlooked problems in sub-Saharan Africa? Diabetes Metab Syndr 2017;11 Suppl 2:S1047–S1051. 10.1016/j.dsx.2017.07.039 28789834

[51] Saeed MEM, Cao J, Fadul B, et al A five-year survey of cancer prevalence in Sudan. Anticancer Res 2016;36:279–86. 26722054

[52] Saeed IE, Weng H-Y, Mohamed KH, et al Cancer incidence in Khartoum, Sudan: first results from the cancer registry, 2009-2010. Cancer Med 2014;3:1075–84. 10.1002/cam4.254 24821265 PMC4303176

[53] Muddathir AM, Kordofani AA, Fadl-Elmula IM Frequency of Bcr-Abl fusion transcripts in Sudanese patients with chronic myeloid leukemia using real-time reverse transcription-polymerase chain reaction. Saudi Med J 2013;34:29–33. 23299156

[54] Kassebaum NJ, Arora M, Barber RM, et al Global, regional, and national disability-adjusted life-years (DALYs) for 315 diseases and injuries and healthy life expectancy (HALE), 1990-2015: a systematic analysis for the global burden of disease study 2015. Lancet 2016;388:1603–58. 10.1016/S0140-6736(16)31460-X 27733283 PMC5388857

[55] Kassebaum N, Kyu HH, Zoeckler L, et al Child and adolescent health from 1990 to 2015: findings from the global burden of diseases, injuries, and risk factors 2015 study. JAMA Pediatr 2017;171:573–92. 10.1001/jamapediatrics.2017.0250 28384795 PMC5540012

[56] Beck S, Wojdyla D, Say L, et al The worldwide incidence of preterm birth: a systematic review of maternal mortality and morbidity. Bull World Health Organ 2010;88:31–8. 10.2471/BLT.08.062554 20428351 PMC2802437

[57] Bashir AO, Ibrahim GH, Bashier IA, et al Neonatal mortality in Sudan: analysis of the Sudan household survey, 2010. BMC Public Health 2013;13:287 10.1186/1471-2458-13-287 23547797 PMC3635916

[58] Federal Ministry of Health, Ministry of Health, Government of South Sudan, Central Bureau of Statistics Southern Sudan Commission of Census S & E Sudan household health survey 2nd round 2010 summary report July; 2010.

[59] Mahfouz MS, Gaffar AM, Bani IA Iodized salt consumption in Sudan: present status and future directions. J Health Popul Nutr 2012;30:431–8. 23304909 PMC3763614

[60] Hussein IS, Min Y, Ghebremeskel K, et al Iodine status and fish intake of Sudanese schoolchildren living in the red sea and white Nile regions. Public Health Nutr 2012;15:2265–71. 10.1017/S1368980012000833 22475452 PMC10271310

[61] Nyuar KB, Min Y, Ghebremeskel K, et al Milk of northern Sudanese mothers whose traditional diet is high in carbohydrate contains low docosahexaenoic acid. Acta Paediatr 2010;99:1824–7. 10.1111/j.1651-2227.2010.01940.x 20618167

[62] Crawford MA, Broadhurst CL, Cunnane S, et al Nutritional armor in evolution: docosahexaenoic acid as a determinant of neural, evolution and hominid brain development. Mil Med 2014;179(11 Suppl):61–75. 10.7205/MILMED-D-14-00246 25373088

[63] Morse NL Benefits of docosahexaenoic acid, folic acid, vitamin D and iodine on foetal and infant brain development and function following maternal supplementation during pregnancy and lactation. Nutrients 2012;4:799–840. 10.3390/nu4070799 22852064 PMC3407995

[64] Ali NI, Elgak SNA, Abdallah YMY, et al Assessment of iodine supplementation program on thyroid function in Sudan. Diabetes Metab Syndr 2019;13:678–80. 10.1016/j.dsx.2018.11.036 30641788

